# Digitally enabled patients, professionals and providers: making the case for an electronic health record in mental health services

**DOI:** 10.1192/pb.bp.115.052191

**Published:** 2016-10

**Authors:** Jonathan Richardson, Joe McDonald

**Affiliations:** 1 Northumberland, Tyne and Wear NHS Foundation Trust, UK

## Abstract

The move to a digital health service may improve some components of health systems: information, communication and documentation of care. This article gives a brief definition and history of what is meant by an electronic health record (EHR). There is some evidence of benefits in a number of areas, including legibility, accuracy and the secondary use of information, but there is a need for further research, which may need to use different methodologies to analyse the impact an EHR has on patients, professionals and providers.

Imagine a digitally enabled health service. The general practitioner (GP) emails the provider a referral letter and copies in the patient, including a link to a website. The patient clicks the link, which takes them to their chosen healthcare provider's landing page. The website contains information about the outcomes of the service from both a patient's and a clinician's perspective and a set of choices about how the patient would like to communicate: by telephone, email, second class post etc. The patient opens an account with their provider, enters all their demographic data (age, ethnic background etc.) and completes whatever screening assessment the service requires. They can also pre-populate their record with some detail about their condition. A digitally enabled professional, working remotely on their telehealth-enabled laptop, has a view of the GP's referral letter and the patient-entered history, and they can then make a decision about what options to offer the patient: a telephone assessment, a telehealth consultation, a face-to-face consultation, an automated online therapeutic intervention, or a referral to an online self-help community.

Many industries have been transformed by computers, but healthcare has not. We have not yet fully enabled self-care for patients; we do not routinely communicate with patients or carers via email but instead rely on insecure and expensive post. We have transferred our paper processes into an electronic format, but are digitally handicapping clinicians. Taking away a highly efficient clinical technology, hand-writing, and replacing it with typing caused the previously administrative tasks to shift on to clinical staff, causing great inefficiency.

However, there are healthcare services that are moving toward a digitally enabled model. For example, the Lloyds Pharmacy Online Doctor (formerly, Dr Thom), a primary care organization, provides a general practice service using existing technologies: the internet, email, text messages and Skype.^1^ Mental health providers should aim to do the same by promoting digitally enabled clinicians.

While developing digitally enabled services we have to be mindful, however, that not every patient and professional has the same digital abilities; part of the general public have limited real-time access to the internet and are new to the use of information technology – they are the so-called ‘digital immigrants’.^2^ This group of patients may lose out if we are not careful. The technological advances such as the internet, social media and smartphones may result in a perceptual change in our patients of what it means to be a healthcare professional in the 21st century,^3^ so we have to be aware of what could be gained (and lost) during the transition to a fully digitally enabled health service.

## Patient health record

The vision of digitally enabled health services is built on the use of information, its documentation and subsequent communication; an individual electronic health record (EHR) is a prerequisite to this vision.

Hippocrates gave a description of a health record, which should serve two functions: ‘A medical record should accurately reflect the course of disease. [It] should indicate the probable cause of disease’.^4^ These two general principles have been built upon over the years. To ensure that an accurate course of a patient's illness is recorded, what is required is not only a documented narrative but also something which will identify the patient as unique. The standardisation of the composition of the record can help to ensure a systematic approach to diagnosis.

The first set of individual case notes is traced back to the US Mayo Clinic's ‘unit medical record’ from 1907.^5^ A standardised set of records for psychiatric in-patients was being used in the Maudsley Hospital in the 1920s, with the secondary purpose of helping to train junior doctors and informing research.^6^ The standardisation of records was unpopular among clinicians in the 1920s, as they felt that they should decide what should be included in the written record, in a fluid and organic manner, akin to the process of the actual examination of the patient.^5^ More recently, the UK's Academy of Medical Royal Colleges (AoMRC) released a set of standards for the structure of the clinical record.^7^ Thus, patient record should contain:

- information taken at admission to hospital
- information taken on discharge from hospital
- information on handover between clinical teams within the hospital.

The AoMRC standards are now being implemented within the health service and a number of professional bodies have used them as a basis for profession-specific documents, such as the Royal College of Psychiatrists' Mental Heath Discharge Summary (MHDS).^8^ The MHDS has been found to be a useful set of standards.^9^

To summarise, the patient record, and good record-keeping, are vital to inform, record and communicate patient care.^10^ The importance of, and need for, a patient record has been highlighted as a prerequisite for clinicians to perform their duties; it should encompass a clinical narrative, standard items and, more recently, coded information.^10^ The clinical narrative is a requirement to ensure that clinicians present a holistic account of patients' problems. Standardisation can help to ensure semantic consistency within and between different clinical groups and areas of work, for example between primary and secondary care. The use of coded information enables specific, anonymous parts of the record for secondary use, for example health research and the revalidation of health professionals.

## Drivers for change

The use of computers and computing in health has grown exponentially, with a central feature being the patient or health record. A definition of a personal health record (PHR), as described by Pagliari, is: ‘a collection of important information about your health or the health of someone you are caring for, such as a parent or child, that you actively maintain and update. The information comes from your healthcare provider, and from you.’^11^

The EHR, which has a broader definition than a PHR, is being used in most Western countries, with governmental policies to promote its implementation.^12^ The US Centers for Medicare & Medicaid notes that the EHR ‘allows healthcare providers to record patient information electronically instead of using paper records’.^12^ This practice has been echoed by broader political, economic, social and technological changes in the UK. The passing of the Health and Social Care Act 2012 is leading to unprecedented changes in the National Health Service (NHS),^13^ whereas the publication of the UK government's information strategy, the *Power of Information*,^14^ and more recently, of *Personalised Health and Care 2020: A Framework for Action*^15^ reaffirms the view that the use of information enabled by EHRs is a central driver to the future direction of healthcare delivery in the UK.

A ‘good doctor’ has been described as a health professional who can synthesise incomplete information, deal with uncertainty, manage risk, constantly adapt to change and take responsibility for their actions.^16^ The documentation of these critical factors within an individual's health record can only serve to improve their care.

The EHR system is still in its early phases within the NHS. The policy drivers are only at the point of being implemented, but at the same time most organisations are being asked to improve the value of the services they provide by enhancing the quality of care with resources that at best stay the same and at worst are reduced.

## Benefits and barriers of e-health

The interplay between health and technology has also been described as e-health.^17^ In a systematic overview conducted by Black *et al*,^18^ e-health is categorised into three areas:

- storage, managing and transmission of data
- clinical decision support
- facilitating care from a distance.

The findings of Black *et al*^18^ are comparable with the initial conclusion drawn by Eysenbach,^17^ namely that there is some evidence that an EHR can lead to improved efficiency, which in turn could improve a Darzi quality measure: clinical effectiveness. There are compelling suggestions that the potential of the EHR lies more within the realm of its secondary use, especially in terms of research linkages, for instance the potential for data prediction and stratification.^19^

The potential to empower patients to make choices would hopefully be enhanced by digital access to their own health information;^15^ however, we need to take into account the current evidence base and ‘lack of clarity’ on how EHRs are supposed to achieve that.^20^

It would seem that the limited evidence of the main constraint of an EHR, increasing the time it takes for clinicians to document information,^21^ could have an adverse impact on the patient–professional interaction, and consequently, on another Darzi quality measure: patient experience. The increased time to document that clinicians report may be due to the numerous NHS regulatory requirements as much as the process of using an EHR, both of which contribute to the data burden experienced by organisations and clinicians.^22^ If the data burden could be reduced it should ultimately improve patient care by freeing up time for clinicians, which they can then spent with patients. Conversely, if EHR data quality is to be of a high enough standard for its secondary research use, what may be required is more time to train clinicians to use an EHR correctly.^23^ This means that there will be a constant balancing, framing and reframing^24^ of the primary and secondary uses of EHRs within health services, a process that needs to be benefits led, with benefits that are clear for patients, professionals and organisations at the outset.

## Use of EHR in health research: examples

A review by Jensen describes a number of potential benefits of using an EHR in secondary research, for example in the field of genetics:^19^ knowledge discovery in databases (KDD) methods by correlating clinical features, predictions from the EHR data, and patient stratification. EHR data could then be linked to the molecular level, such as in pharmacogenomics and gene network-based decision support.^19^

An example of good practice in this area in mental health services is the Clinical Record Interactive Search (CRIS)^20^ system used by a number of mental health trusts that provides access to anonymised clinical data for researchers in a robustly governed manner.

The use of records to create linkage within the EHR data has also been recognised and led to the establishment of the Centre for Health Service and Academic Partnership in Translational E-Health Research. This is one of four e-health informatics research centres, and one of the central strands to the UK government's Life Sciences Strategy.^25^

Future research should take account of the ‘political, pragmatic and commercial drivers of the decision-making in the commissioning of e-health’,^18^ which in turn need to inform a paradigm shift in the approach to research, for example, changing the focus toward a value- and outcome-based approach.^26^

## Implications for the future

If one considers the implications of adopting any scientific or technical system, in this case information management and technology, in a social system, in this case medicine, and the benefits and barriers which may characterise both systems, then a fuller understanding of a ‘socio-technical’ approach to the EHR implementation may emerge.^27,28^

The socio-technical approach is already starting to take root in the NHS and will affect practice; one major premise is that there is no such thing as an IT project and we should articulate that any change needs to be care quality led and clinically based; IT should enable the change and not drive it. One example is assessing how usable systems are; the mental health informatics community has been an advocate of usability testing and working in partnership with EHR suppliers.^29^ If working with industry and the use of industry standard usability scales^30^ become the norm, this may lead to a new approach in future implementations. For example, we could see joint ventures between NHS trusts and EHR vendors, with EHRs implemented at a local level using national, professionally agreed standards.

The national research programme to evaluate the effect of IT on patient care has produced a summary report evaluating the results of the National Programme for IT.^31^ This was a substantially funded and ambitious research programme that recognised that traditional methods of evaluation may not be appropriate for the evaluation of health informatics. Seeing that an EHR has a complicated research base, a meta-narrative approach was suggested as it can systemically make sense of ‘complex, heterogeneous and conflicting bodies of literature’.^32^ It does so by attempting to deconstruct the research according to underlying philosophical positions (positivist, interpretivisit, critical and recursive) and linking this to the historical roots of EHR research. The latter are evidence-based medicine, human–computer interaction, workplace design, symbolic interactionism, safety-critical systems, social practice view of knowledge, complexity theory and the philosophy of science. Greenhalgh *et al*^32^ describe a number of areas where further research is needed: theory-building, collaborative clinical work, how EHR systems should be co-designed, differences between ‘off the shelf’ and ‘home-grown’ EHRs, the ethics of data-sharing, reviews of implementation within organisations, and knowledge translation between what is known and assumed. The authors also suggested three areas where research apparently is not needed: simplified experimental designs, surveys of staff attitudes which are not contextualised, and under-theorised qualitative research of failed implementations.

Information, communication and documentation are vital components of a future vision of digitally enabled patients, professionals and providers. We need to move from imagining a digitally enabled health service to implementing it; some organizations are already using digital enablers such as mobile working and digital dictation with their clinicians.^33^ The Health Foundation has looked at the implications of Learning Healthcare Systems and Foley & Fairmichael suggest that ‘structured electronic health records, rigorous outcome measurement and alternative research methodologies offer the possibility of a healthcare system that learns from each patient who is treated’.^34^

One of the most important drivers for a digitally enabled health service will be the patients themselves. Patients are the experts in their own health issues. The technology that patient groups are already adopting, such as online patient communities (e.g. ACOR.org),^35^ to help them better understand, manage and deal with their health issues, may in turn inform the understanding of how, where and why digital services are used. Attempts have been made to ensure that health and wellness apps are designed to recognised standards.^36^ The next step is to ‘let patients help steer our decisions, strategic and practical. Let patients define what value is’,^35^ so that we can delineate the true purpose, and recognise the challenge, of the NHS developing meaningful digitally enabled services for patients, professionals and providers.
